# α-Glucosidase inhibition by flavonoids: an *in vitro* and *in silico* structure–activity relationship study

**DOI:** 10.1080/14756366.2017.1368503

**Published:** 2017-09-21

**Authors:** Carina Proença, Marisa Freitas, Daniela Ribeiro, Eduardo F. T. Oliveira, Joana L. C. Sousa, Sara M. Tomé, Maria J. Ramos, Artur M. S. Silva, Pedro A. Fernandes, Eduarda Fernandes

**Affiliations:** a UCIBIO, REQUIMTE, Laboratory of Applied Chemistry, Department of Chemical Sciences, Faculty of Pharmacy, University of Porto, Porto, Portugal;; b UCIBIO, REQUIMTE, Faculty of Sciences, Department of Chemistry and Biochemistry, University of Porto, Porto, Portugal;; c Department of Chemistry & QOPNA, University of Aveiro, Aveiro, Portugal

**Keywords:** Diabetes, flavonoids, α-glucosidase inhibition, *in vitro*, *in silico*

## Abstract

α-Glucosidase inhibitors are described as the most effective in reducing post-prandial hyperglycaemia (PPHG) from all available anti-diabetic drugs used in the management of type 2 diabetes *mellitus*. As flavonoids are promising modulators of this enzyme’s activity, a panel of 44 flavonoids, organised in five groups, was screened for their inhibitory activity of α-glucosidase, based on *in vitro* structure–activity relationship studies. Inhibitory kinetic analysis and molecular docking calculations were also applied for selected compounds. A flavonoid with two catechol groups in A- and B-rings, together with a 3-OH group at C-ring, was the most active, presenting an IC_50_ much lower than the one found for the most widely prescribed α-glucosidase inhibitor, **acarbose**. The present work suggests that several of the studied flavonoids have the potential to be used as alternatives for the regulation of PPHG.

## Graphical abstract

**Figure:**
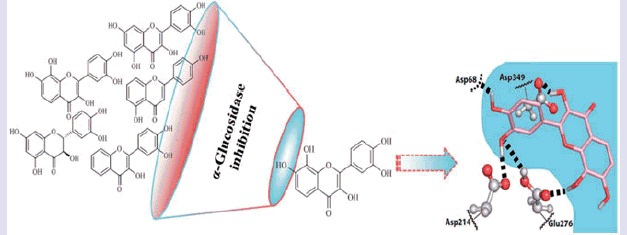

## Introduction

Diabetes mellitus (DM) is one of the most significant public health concerns worldwide. According to the International Diabetes Federation, there are 415 million of adults with diabetes, probably reaching 642 million in 2040^1^. DM is a multifactorial metabolic disorder, characterised by chronic hyperglycaemia and can be primarily classified as type 1 (insulin dependent DM) and type 2 (non-insulin dependent DM)^2^. Type 2 DM is the most common form of DM, accounting for more than 90% of all diabetic patients, and results from the interaction between behavioural, environmental and genetic risk factors^3^ ^,^ ^4^. Diabetic patients are more vulnerable to various forms of both short- and long-term complications, such an increased risk of common infections and cancer, increasing the morbidity and mortality^4–6^.

DM is characterised by absolute or relative deficiency in insulin secretion by pancreatic β-cells, increased insulin resistance and/or impairment of insulin action in target tissues^7–10^. Post-prandial hyperglycaemia (PPHG) is known as the plasma glucose value taken 1.5–2 h after a meal. The disequilibrium in the insulin regulatory system, culminates in high PPHG level (≥200 mg/dL) compared to individuals without type 2 DM (<140 mg/dL)^11^. Prolonged hyperglycaemia has been associated with several macro- and micro-vascular complications, e.g. nephropathy, retinopathy and cardiovascular disorders^11–13^. Therefore, the management of PPHG, in order to achieve blood glucose levels as close to normal as possible, is considered by the scientific community one of the major therapeutic strategies for type 2 DM treatment^14^.

An effective control of hyperglycaemia in type 2 DM includes the retarding, regulation and/or inhibition of carbohydrate hydrolysing enzymes^15^. Among these, α-glucosidase, which is located in the brush border of the enterocytes of the jejunum, is the most important enzyme in carbohydrates digestion^16^. It catalyses the hydrolysis of 1,4-α bonds of the unabsorbed oligo- and disaccharides, and converts them into monosaccharides, namely, glucose, which are absorbed in the upper jejunum, resulting in hyperglycaemia^17^ ^,^ ^18^. Inhibitors of α-glucosidase can retard the decomposition and absorption of dietary carbohydrates by restricting the breakdown of linear or branched oligosaccharide units like α-limit dextrins, maltose and maltotriose to produce glucose, thereby preventing glucose absorption into blood stream and suppress the PPHG^19^ ^,^ ^20^.

The use of α-glucosidase inhibitors started in the 1970s and the first related therapeutic agents for diabetes were approved in the 1990s^18^. Nowadays, several oral drugs are used such as voglibose, acarbose and miglitol to inhibit the α-glucosidase activity^20^. However, their use has been associated with several adverse effects such as abdominal distention, flatulence, diarrhoea and pneumatosis cystoides intestinalis^11^. Most of these side-effects occur when undigested carbohydrates are fermented by colonic bacteria^21^ ^,^ ^22^. The commonly used α-glucosidase inhibitors were also reported to be associated with rare adverse hepatic events^23^. Moreover, these drugs have low efficacy, with high IC_50_ values^24^ ^,^ ^25^. Thus, much effort has been undertaken to search for more effective and safer inhibitors.

Some flavonoids, such as morin^17^, luteolin^26^, baicalein^27^, kaempferol^28^ and apigenin^24^, have been shown to exhibit inhibitory effects against α-glucosidase enzymes. Nevertheless, the information found in the literature is disperse and variable, probably due to the use of different enzyme concentrations (0.3–1.7 U/mL)^8^ ^,^ ^29^ ^,^ ^30^, from different origins (e.g. yeast and rat intestinal α-glucosidase)^18^ ^,^ ^19^ ^,^ ^31^ ^,^ ^32^, different substrates concentrations (1–20 mM)^19^ ^,^ ^30^ ^,^ ^33^ and incubation time with the flavonoids (5 min–3 h)^8^ ^,^ ^26^ ^,^ ^28^ ^,^ ^29^ ^,^ ^34^. The discrepancies between the studies bring difficulties to their comparison and consequently the conclusions about the flavonoids efficacy against α-glucosidase activity.

**Figure 1.**
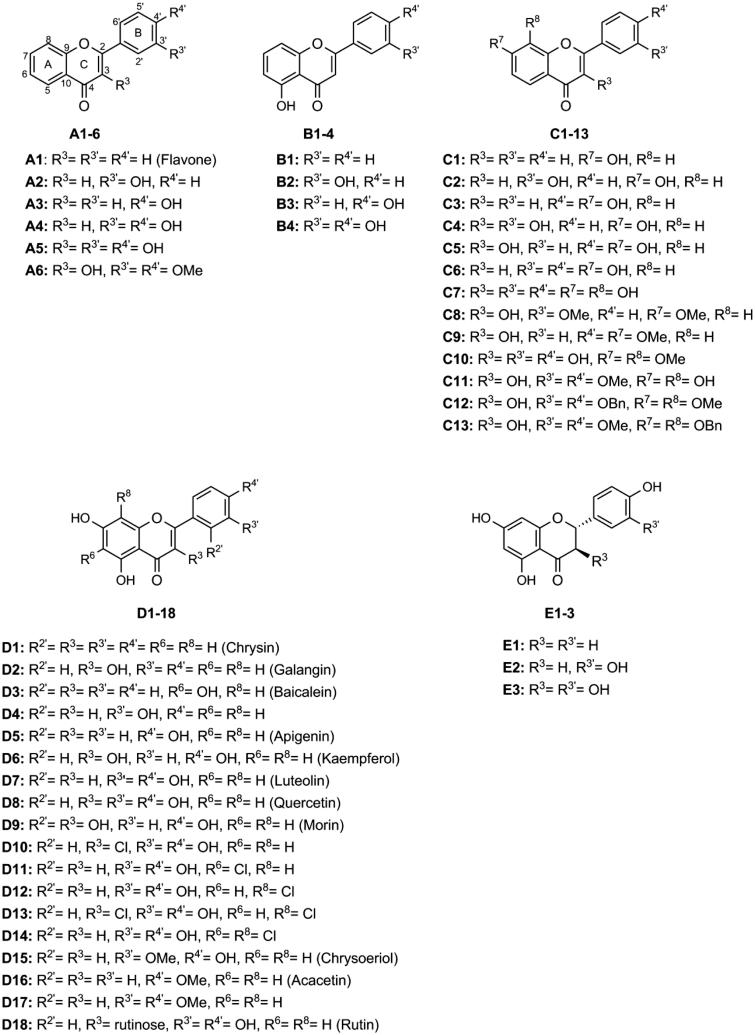
Chemical structures of the tested flavonoids.

The present study aims to establish a structure–activity relationship of a panel of flavonoids (Figure 1), divided in five groups, against α-glucosidase activity, covering a solid set of different structures. For this purpose, a microanalysis screening system was applied for testing the type of inhibition of the most active flavonoids from each group, and the positive control **acarbose**. Molecular docking calculations were also applied to complement the inhibitory activity studies and to predict the binding model of the selected flavonoids to the three-dimensional structure of α-glucosidase.

The overall obtained results constitute an important step for the establishment of the adequate and ideal chemical structure of flavonoids to target α-glucosidase and consequently, to manage type 2 DM.

## Materials and methods

### Chemicals

The following reagents were purchased from Sigma-Aldrich Co. LLC (St. Louis, MO): α-glucosidase from *Saccharomyces cerevisiae*, *p*-nitrophenyl-α-D-glucopyranoside (*p*NPG), DMSO, NaHPO_4_, Na_2_HPO_4_, compounds **D3** (baicalein), **D4** (apigenin), **D6** (kaempferol), **D7** (luteolin), **D8** (quercetin), **D9** (morin), **D16** (acacetin), **D18** (rutin), **E1** (naringenin), **E2** (eriodictyol), **E3** (taxifolin) and **acarbose**. Compounds **A1**, **A2**, **A3**, **A4**, **A5**, **A6**, **B1**, **B2**, **B3**, **B4**, **C1**, **C2**, **C3**, **C4**, **C5**, **C6**, **C8**, **C9**, **D1** (chrysin), **D2** (galangin) and **D5** were obtained from Indofine Chemical Company, Inc. (Hillsborough, NJ). The flavonoids **C7**, **C10**, **C11**, **C12**, **C13**, **D10**, **D11**, **D12**, **D13**, **D14** and **D17** were synthesised as previously described^35–37^. For the enzymatic assays, flavonoids were dissolved in DMSO (the final concentration of DMSO in the reactional mixture was 4.76%).

### *In vitro* inhibition assay for α-glucosidase activity

The α-glucosidase activity was measured using a method of Tadera et al.^31^ slightly modified. The assay was carried out by monitoring the α-glucosidase-mediated transformation of the substrate *p*NPG into α-D-glucose and *p*-nitrophenol, at 405 nm. Briefly, in a 96-well plate, the enzyme (0.05 U/mL), dissolved in 100 mM phosphate buffer (pH 6.8), was pre-incubated at 37 °C for 5 min with the flavonoids (0–200 μM). In sequence, *p*NPG was added (600 μM) and incubated in the reaction mixture at 37 °C for 30 min. The enzymatic reaction was monitored spectrophotometrically, in a microplate reader (Synergy HT, BIO-TEK), by measuring the absorbance at 405 nm. The obtained values correspond to the slope measured between 5 and 20 min. The amount of DMSO used had no interference with the assay. Acarbose (0–2000 μM) was used as positive control. The obtained results represent at least three independent experiments.

### Inhibitory kinetic analysis

The inhibitory kinetic analyses were performed for the most active flavonoids of each group (A–E): **A5** (0–62.5 μM), **B3** (0–50 μM), **C7** (0–5 μM), **D8** (quercetin) (0–12.5 μM), **D10** (0–18.75 μM), **E3** (taxifolin) (0–100 μM) and for the positive control, **acarbose** (0–2000 μM).

Briefly, the enzyme (0.05 U/mL), dissolved in 100 mM phosphate buffer (pH 6.8), was pre-incubated at 37 °C with the above mentioned flavonoids in a 96-well plate, for 5 min. In sequence, *p*NPG was added (300, 600 and 1200 μM) and incubated in the reaction mixture at 37 °C for 30 min.

The study of the inhibition type (competitive, uncompetitive, non-competitive or mixed) of the tested flavonoids was performed using the nonlinear regression Michaelis–Menten enzyme kinetics and the corresponding Lineweaver–Burk double reciprocal plots for each concentration of the inhibitor and substrate. The Ki values were calculated with GraphPad Software 6.0 by plotting the reciprocal of maximum velocity (1/*V*_max_) (*y* axis) against the flavonoid concentrations (*x* axis) (Table 2)^38–40^.

The type of inhibition parameters were all calculated with GraphPad Software 6.0. The obtained results represent at least three independent experiments.

### Molecular docking

#### Homology modelling

3D structures of *Saccharomyces cerevisiae* α-glucosidase (maltase, EC 3.2.1.20) are unavailable. There are, however, X-ray structures available for the isozyme isomaltase/α-methylglucosidase (EC 3.2.1.10). The isomaltase/α-methylglucosidase with the PDB^41^ entry 3AJ7 has a high-resolution X-ray structure and a high sequence identity (72.51%) and sequence similarity score (0.54) with the *Saccharomyces cerevisiae* α-glucosidase MAL32 (UniProt entry P38158)^42^.

We identified several differences between residues lining the binding pockets of α-glucosidase and isomaltase, such as the pairs Asp307/Glu204, Asp408/Glu411, Phe157/Tyr158, Thr215/Val216, Ala278/Gln279, Val303/Thr306 or Ala178/Cys179, respectively for α-glucosidase and isomaltase. Most of these differences involve very similar residues, but considering the differences in the size of the side chains (which can affect binding through stereochemical effects) and the high confidence entrusted by the extremely favourable sequence identity, we opted to build a high accuracy homology model of α-glucosidase using isomaltase as template.

Using MAL32 target sequence and the structure with the PDB ID 3AJ7 as template, we built the α-glucosidase homology model in SWISS-MODEL^43^ based on the target-template alignment using ProMod3. The resulting model has a Global Model Quality Estimation of 0.92 and a rmsd for 4452 atoms of 0.40 Å, which is excellent. Supplementary Figure S1 provides a summary of the homology modelling parameters and Supplementary Figure S2 the Ramachandran plot analysis of the homology model.

#### Docking protocol

The protonation states of the enzyme were predicted using the Protoss server^44^. Accordingly, His6, His97 and His251 were modelled in the charged form, Glu276 and Glu481 were modelled in the neutral form. All other residues were protonated according to their p*K*_a_ at pH 7.

The substrate α-maltose was modelled inside the binding pocket and subsequently, to remove potential clashes and relax the structure of the model, Molecular Mechanics minimisation followed by classical MD simulations were done. This protocol was performed within the AMBER 12^45^ suite using the ff99SB^46^ force field for the protein and GLYCAM06^47^ force field for maltose. The details for the energy minimisation and MD simulation are given in SI.

To validate the receptor for the docking protocol we extracted the last step from the MD simulation and re-docked the substrate α-maltose and also the inhibitor acarbose. Since the predicted binding modes, both for α-maltose and acarbose^48^, behaved according to the expected catalytic binding mode and to the X-ray structure 3TOP, we proceeded to binding mode prediction of the most active flavonoids of each group (A–E).

AutoDock Vina^49^ was used to generate binding poses using default parameters (see Supplementary Material for details), such as the exhaustiveness of the global search = 8 or the maximum number of output poses = 9. The search space was confined to a box of circa 15 × 20 × 17 centred on the site occupied by the second glucose in maltose. The receptor was treated as a rigid body and prepared using openbabel^50^ for atom-typing and charge assignment using Gasteiger–Marsili charges^51^. Flavones atom-types and charges were attributed the same way.

Subsequently, the poses generated by AutoDock Vina were rescored using the scoring function ChemPLP, which is excellent for oligosaccharides^52^. We used the GOLD software^53^ with the standard parameters for a “Rescoring Run” with local optimisation and selected the binding mode with the highest score.

## Results

### *In vitro* inhibition assay for α-glucosidase activity

The inhibitory effect of flavonoids against α-glucosidase activity is shown in Table 1. This effect was highly dependent on small variations of the flavonoids’ structures. According to the flavonoids’ structures, we divided the studied compounds into five groups (A–E).

**Table 1.** Structures and *in vitro* α-glucosidase inhibition by the studied flavonoids (IC_50_ μM, mean ± SEM).

Compounds	Structure	R^2′^	R^3^	R^3′^	R^4′^	R^6^	R^7^	R^8^	IC_50_ (μM)
**A1** (Flavone)		–	H	H	H	–	–	–	<20%*200 μM^a^
**A2**		–	H	OH	H	–	–	–	<20%*200 μM^a^
**A3**		–	H	H	OH	–	–	–	<20%*200 μM^a^
**A4**		–	H	OH	OH	–	–	–	32 ± 4%*200 μM^a^
**A5**		–	OH	OH	OH	–	–	–	**54 ± 3**
**A6**		–	OH	OMe	OMe	–	–	–	<20%*100 μM^a^
**B1**		–	–	H	H	–	–	–	<20%*200 μM^a^
**B2**		–	–	OH	OH	–	–	–	31 ± 4%*200 μM^a^
**B3**		–	–	H	OH	–	–	–	**66 ± 2**
**B4**		–	–	OH	OH	–	–	–	**66 ± 7**
**C1**		–	H	H	H	–	OH	H	<20%*200 μM^a^
**C2**		–	H	OH	H	–	OH	H	53 ± 4
**C3**		–	H	H	OH	–	OH	H	≈200
**C4**		–	OH	OH	H	–	OH	H	42 ± 4
**C5**		–	OH	H	OH	–	OH	H	96 ± 10
**C6**		–	H	OH	OH	–	OH	H	95 ± 7
**C7**		–	OH	OH	OH	–	OH	OH	**7.6 ± 0.4**
**C8**		–	OH	OMe	H	–	OMe	H	22 ± 2%*100 μM^a^
**C9**		–	OH	H	OMe	–	OMe	H	<20%*200 μM^a^
**C10**		–	OH	OH	OH	–	OMe	OMe	86 ± 6
**C11**		–	OH	OMe	OMe	–	OH	OH	31 ± 3%*200 μM^a^
**C12**		–	OH	OBn	OBn	–	OMe	OMe	<20%*200 μM^a^
**C13**		–	OH	OMe	OMe	–	OBn	OBn	32 ± 3%*200 μM^a^
**D1** (Chrysin)		H	H	H	H	H	–	H	<20%*50 μM^a^
**D2** (Galangin)		H	OH	H	H	H	–	H	21 ± 3%*200 μM^a^
**D3** (Baicalein)		H	H	H	H	OH	–	H	44 ± 3
**D4**		H	H	OH	H	H	–	H	89 ± 3
**D5** (Apigenin)		H	H	H	OH	H	–	H	82 ± 6
**D6** (Kaempferol)		H	OH	H	OH	H	–	H	32 ± 3
**D7** (Luteolin)		H	H	OH	OH	H	–	H	46 ± 6
**D8** (Quercetin)		H	OH	OH	OH	H	–	H	**15 ± 3**
**D9** (Morin)		OH	OH	H	OH	H	–	H	32 ± 2
**D10**		H	Cl	OH	OH	H	–	H	21 ± 2
**D11**		H	H	OH	OH	Cl	–	H	≈200
**D12**		H	H	OH	OH	H	–	Cl	55 ± 2
**D13**		H	Cl	OH	OH	H	–	Cl	43 ± 3
**D14**		H	H	OH	OH	Cl	–	Cl	34 ± 3
**D15** (Chrysoeriol)		H	H	OMe	OH	H	–	H	156 ± 5
**D16** (Acacetin)		H	H	H	OMe	H	–	H	≈200
**D17**		H	H	OMe	OMe	H	–	H	<20%*200 μM^a^
**D18** (Rutin)		H	Rutinose	OH	OH	H	–	H	<20%*200 μM^a^
**E1** (Naringenin)		–	H	H	–	–	–	–	45 ± 3%*200 μM^a^
**E2** (Eriodictyol)		–	H	OH	–	–	–	–	35 ± 4%*200 μM^a^
**E3** (Taxifolin)		–	OH	OH	–	–	–	–	**≈200**
Positive control: **Acarbose**		–	–	–	–	–	–	–	607 ± 56

a Inhibitory activity (mean ± SEM %) at the highest tested concentration (in superscript).

**Table 2.** *K*_i_ values (mean ± SEM, μM) for the inhibition of yeast α-glucosidase by the selected flavonoids.

Flavonoid	Type of inhibition	*K*_i_ (μM)
**A5**	Mixed	41 ± 7
**B3**	Mixed	127 ± 9
**C7**	Competitive	6.5 ± 0.2
**D8** (quercetin)	Competitive	6.8 ± 0.6
**D10**	Non-competitive	51 ± 6
**E3** (taxifolin)	Non-competitive	347 ± 15
Positive control: **Acarbose**	Competitive	457 ± 11

#### Flavonoids of A group

From all the flavones from A group, flavone **A5** was noticeably the most effective, presenting an IC_50_ of 54 ± 3 µM. For the other tested compounds, it was not possible to achieve the IC_50_ value up to the concentrations of 100 µM (**A6**) or 200 µM (**A1**–**A4**). These results indicate that the presence of a 3-OH group in the C-ring is relevant for the inhibitory activity of flavonoids. Moreover, when comparing the flavonoid **A5** and the flavonoid **A6**, we may conclude that the replacement of the 3′,4′-(OH)_2_, in the B-ring, by methoxy (–OMe) groups withdraws the inhibitory activity of compound **A5**.

#### Flavonoids of B group

From B group, flavone **B1** was the less active. Comparing the activities of **B1** (with a 5-OH in A-ring) and **A1** (without substitutions) it is shown that the presence or absence of 5-OH in A-ring, *per se*, is indifferent for the inhibitory activity. However, the 5-OH substitution in A-ring, together with the 4′-OH (**B3**) or 3′,4′-(OH)_2_ (**B4**) in B-ring, increases the activity, comparing with the related compounds, from A group, which do not have 5-OH substitution.

#### Flavonoids of C group

C group has the most active flavonoid, **C7** with an IC_50_ = 7.6 ± 0.4 µM, which is almost 80 times lower than the IC_50_ of the positive control, **acarbose** (IC_50_ = 607 ± 56 µM). Comparing the flavones **C5** and **C4**, it is clear that the presence of –OH groups in the B-ring is more relevant in the 3′-position (**C4**, IC_50_ = 42 ± 4 µM) than in the 4’-position (**C5**, IC_50_ = 96 ± 10 µM). Flavone **C1**, with just one 7-OH group, did not achieve the IC_50_ value up to the maximum tested concentration (200 µM). However, adding another 3′-OH at the B-ring (**C2**) is enough to increase the inhibitory activity of the compound (IC_50_ = 53 ± 4 µM). In turn, increasing the complexity of the structure by the addition of a –OMe and/or a benzyloxy (–OBn) group (**C8**–**C13**) did not favour the activity.

#### Flavonoids of D group

In what concerns D group, the most effective compound was **D8** (quercetin) with an IC_50_ = 15 ± 3 µM. The substitution in 3-position of the C-ring seems to be advantageous for the intended activity, since **D7** (luteolin), with no substitution in 3-position, had a higher IC_50_ (46 ± 6 µM). Moreover, the relevance of the existence of a 3-OH substitution in the C-ring was evidenced by the low activity of **D18** (rutin), which had a rutinose in this position. In turn, the close IC_50_ values of **D8** (quercetin) and **D10** (IC_50_ = 21 ± 2 μM) indicate that the presence of a 3-OH group or a 3-Cl atom in the C-ring is indifferent for α-glucosidase inhibition. The relevance of the presence of a catechol moiety in the B-ring is shown by the different IC_50_ values found for **D7** (luteolin) (46 ± 6 µM), which have a catechol group in 3′,4′- positions, and the compounds **D5** (apigenin) (IC_50_ = 82 ± 6 µM) and **D4** (IC_50_ = 89 ± 3 µM), which had just one OH in the 4′- or 3′-positions, respectively. Similarly to the obtained results in C group, results of D group indicate that the methylation of the catechol arrangement in the B-ring (**D15**–**D17**) did not show any advantage for the studied activity.

#### Flavonoids of E group

Analysing the IC_50_ of flavonoids from E group, **E3** (taxifolin) had the best inhibitory activity. Nonetheless, its IC_50_ was approximately 200 μM, the maximum tested concentration.

### Inhibitory kinetics analysis

**Figure 2.**
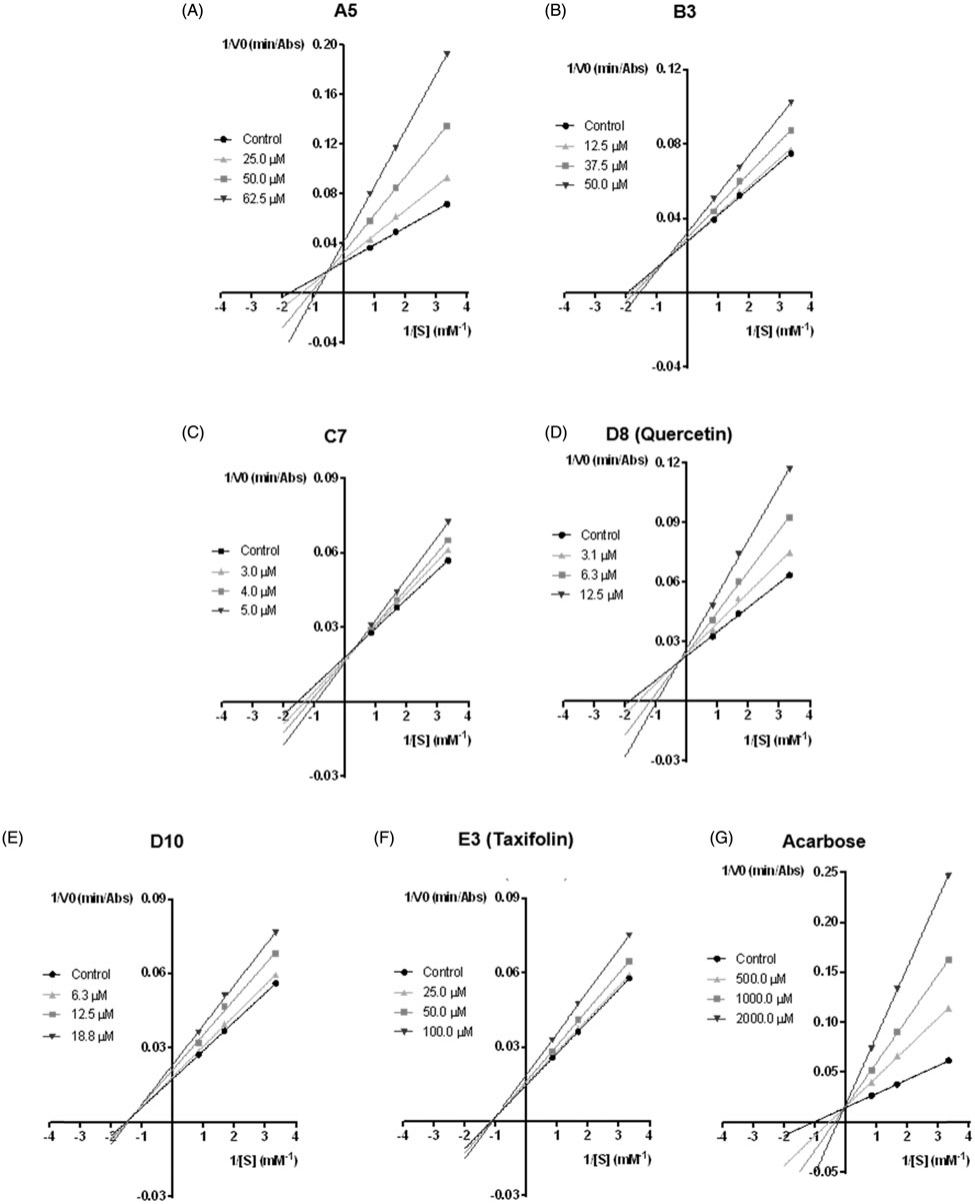
Lineweaver–Burk double reciprocal plots of α-glucosidase inhibition by **A5** (A), **B3** (B), **C7** (C), **D8** (quercetin) (D), **D10** (E), **E3** (taxifolin) (F) and **acarbose** (G).

The type of inhibition of the most active flavonoids of each group (A–E), **A5**, **B3**, **C7**, **D8** (quercetin), **D10**, **E3** (taxifolin) and **acarbose** was deducted from the calculation of Km and maximum enzyme velocity (*V*_max_). These parameters were obtained by the nonlinear regression Michaelis–Menten enzyme kinetics and complemented with Lineweaver–Burk double reciprocal plots. As observed in Figure 2, flavones **A5** (Figure 2(A)) and **B3** (Figure 2(B)) showed a mixed type inhibition. Concerning flavonoid **C7**, although the Figure 2(C) does not clearly differentiate between a mixed and a competitive inhibition type, the interception of the lines is close to the y axis, suggesting a competitive pattern. Flavonoid **D8** (quercetin) (Figure 2(D)) and the positive control **acarbose** (Figure 2(G)) have shown a competitive inhibition type. Flavones **D10** (Figure 2(E)) and **E3** (taxifolin) (Figure 2(F) presented a non-competitive inhibition type, since the *K*_m_ value remained constant and the *V*_max_ value decreased with increasing concentrations of the flavonoids.

The *K*_i_ values were calculated with GraphPad Software 6.0 by plotting the reciprocal of maximum velocity (1/*V*_max_) (*y* axis) against the flavonoid concentrations (*x* axis) (Table 2).

### Binding mode of the selected inhibitors/flavonoids

We have investigated the binding poses of a selection of inhibitors within the reactive pocket of the active site of α-glucosidase [**acarbose**, **A5**, **B3**, **C7**, **D8** (quercetin), **D10** and **E3** (taxifolin)]. It is important to realise that a few of them may bind in more than one sugar pocket, even predominantly in other sugar pockets that line the active site cavity (which were physiologically developed to bind the sugars of oligosaccharide chain tips), and in a lesser extent at the deeper, reactive pocket of the active site. However, for the purpose of drug discovery, the deep reactive pocket is by far the most relevant, because it is the one for which the drugs are always optimised to bind (an evidence of this is that all approved drugs for this target are described as competitive inhibitors). Therefore, it was the binding to this deep pocket that we explore in this section.

#### Acarbose

**Figure 3.**
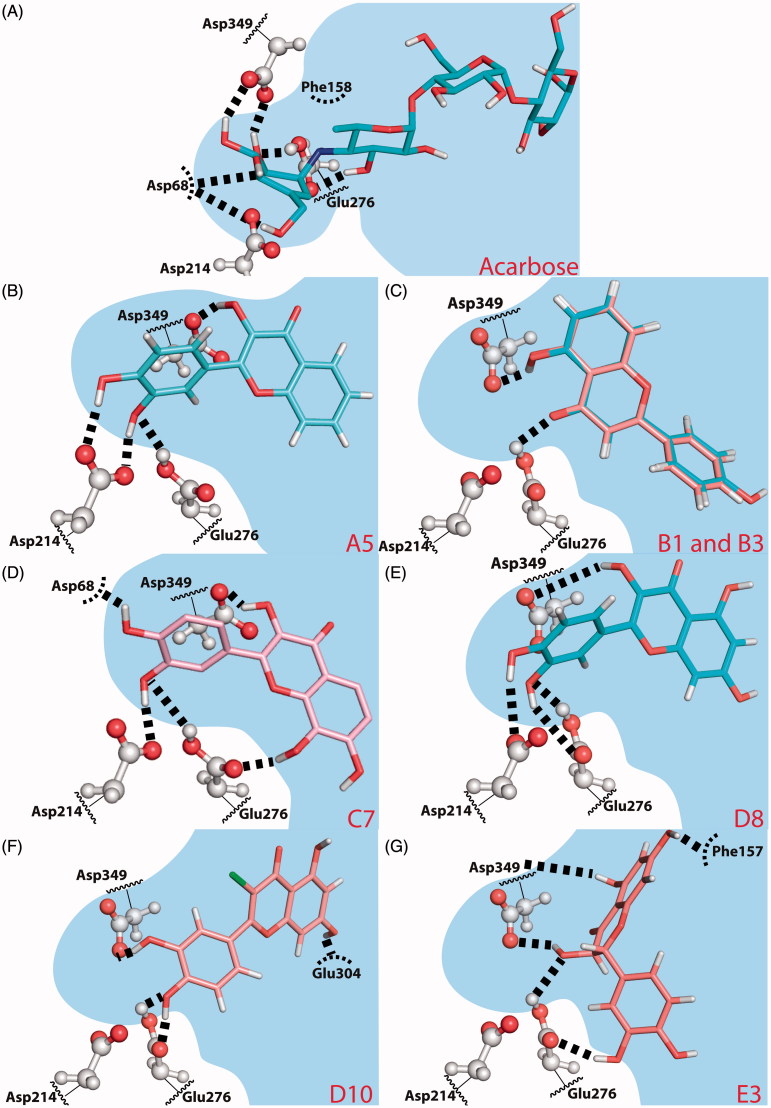
Predicted binding poses for **acarbose** (A) and flavones **A5** (B), **B1** and **B3** (C), **C7** (D), **D8** (quercetin) (E), **D10** (F) and **E3** (taxifolin) (G). Hydrogen bonds are represented by dashed lines. (B) **B1** is coloured salmon and **B3** light blue. The residues that establish the most relevant interactions are also shown. Asp214 and Glu276 are the catalytic residues that participate in the hydrolysis reaction. Asp349 is also a conserved residue.

The predicted binding mode for **acarbose** in the homology model (Figure 3(A)) positions the first glucose of **acarbose** in the exact same site as the first glucose of the α-maltose substrate, which was used to build and refine the model. Thus, the first glucose of **acarbose** is placed in the deep reactive pocket with 2,3,4-(OH)_3_ groups donating hydrogen bonds to Asp349 and Asp68. The oxygen atoms from these three hydroxyls accept hydrogen bonds from Arg212, His348 and Arg439. The single difference between **acarbose** first glucose and α-maltose is in the CH_2_OH group, since in α-maltose the C-4 has sp^3^ hybridisation while in **acarbose** the C-4 has sp^2^ hybridisation. Therefore, the α-maltose CH_2_OH group hydrogen bonds to both His111 and Asp214 while the same group in acarbose makes a single hydrogen bond with Asp68.

The nucleophilic Asp214 is placed below the acarbose C-1 carbon but does not accept any hydrogen bond from **acarbose**. Different to α-maltose binding, Glu276 donates one hydrogen bond to the *N*-glycosidic bond nitrogen and accepts one hydrogen bond from the 3-OH group of the second **acarbose** glucose.

To conclude, the first glucose, the *N*-glycosidic bond and the second glucose of **acarbose**, all occupy the same site as the substrate α-maltose and have a binding mode identical to α-maltose. This is compatible with a competitive mode of inhibition, which the Lineweaver-Burk double reciprocal plots of Figure 2(G) seem to corroborate.

#### Flavone A5

Flavones skeleton is quite different from the saccharides found in α-maltose and **acarbose**. Nevertheless, when we analyse the predicted binding poses for the most active compounds, we can observe similar interactions with binding pocket residues.

In the case of the most active compound of A group, **A5**, the catechol group in the B-ring and the OH substitution in the 3-position of the C ring, provide similar interactions to active site residues as found in α-maltose or **acarbose** (Figure 3(B)). The 3′,4′-(OH)_2_ B-ring substitutions donate hydrogen bonds to Asp214 and the neutral Glu276 donates one hydrogen bond to B-ring 3′-OH. The OH in the 3-position of the C-ring donates a hydrogen bond to the conserved Asp349.

The B-ring binds in the small crevice of the active site pocket, occupying the same site as the first glucose of **acarbose** and α-maltose. The C-ring occupies a similar site to α-maltose second glucose, in the entrance of the deep small pocket, while the A-ring is exposed to the larger binding pocket.

The Lineweaver–Burk double reciprocal plots (Figure 2(A)) shows a mixed inhibition type. The binding mode described here for flavone **A5** corresponds to the competitive inhibition mode. We directed the binding mode prediction to the active site, where the natural substrates bind the first two sugar rings and did not explore all possible modes of interaction of flavones with α-glucosidase, as the interactions with the active site are the most relevant for drug design. However, given the large size of the active site and knowing that many natural substrates are oligosaccharides, it is possible that the inhibitors bind to the sites where the oligosaccharides sugar rings (other than the first two) bind. Therefore, we cannot exclude that flavone **A5** may bind with increased affinity to a different oligosaccharide site, which could interfere with substrate binding and explain the mixed inhibition type.

#### Flavone B3

When flavone **B3** binds the reactive region of the active site it is predicted to bind the A- and C- rings close to the catalytic Glu276 and the conserved Asp349, establishing strong hydrogen bonds with them (Figure 3(C)). However, **B3** does not insert into the deepest region of the catalytic pocket, formed by Asp349/Glu276/Asp214 triad (Asp214 is the nucleophilic residue), but instead in a larger, shallower region of the pocket bordered by hydrophobic residues (such as Phe157, Phe158, Phe177, Thr215 and Phe300) and polar residues (His245, His279, Glu304, Arg312 or Asp408). This binding mode is very similar to the one obtained for flavone **B1** (Figure 3(C)). The additional 3′-OH group of flavone **B3** might hydrogen bond His274, but it is not totally clear from our binding mode prediction.

Despite **B3** binding outside the small crevice formed by the catalytic residues, the predicted mode of binding would still be more compatible with a competitive mode of inhibition. The reason is **B3** C-ring binds exactly in the site of the α-maltose second glucose. The binding pose shown in Figure 3(C) will not allow for substrate binding, despite the fact that the data shown in Figure 2(B) seems to point to the opposite. As mentioned earlier, it is possible that the inhibitors bind to the sites where the oligosaccharides sugar rings (other than the first two) bind. These cases are less relevant than the one shown here for the actual purpose of drug discovery, but could allow for substrate binding even though interfering with the substrate pose.

#### Flavone C7

Comparing the inhibitory activity of **C1** and **C3** with **C6**, it seems that addition of –OH groups to the B-ring improves the inhibitory activity of group C flavonoids, with the 3′-OH substitution to have the greater impact on the inhibitory activity (Table 1). Flavone **C7** has both of these substitutions, plus three more –OH substitutions, one on the C-ring (3-OH) and two on the A-ring (7,8-(OH)_2_).

The predicted binding mode for flavone **C7** in the active site region places the B-ring deep in the small crevice formed by the catalytic residues. Glu276 hydrogen bonds to 3′-OH, and 3′-OH donates a hydrogen bond to Asp214. 4′-OH group is even deeper in the small pocket and hydrogen bonds Asp68.

The C- and A-rings bind in the entrance of the small crevice, with C-ring 3-OH substitution hydrogen bonding Asp349. The A- and C-rings are placed around Glu276, and 8-OH group donates a hydrogen bond to Glu276. The 7-OH substitution is exposed to the larger binding pocket and no specific interactions with 8-OH group can be identified.

Considering this binding mode, the strong hydrogen bond interactions with active site amino acids are consistent with a very favourable binding affinity to the active site, indicating a competitive type of inhibition.

#### Flavones D8 (quercetin) and D10

Figure 2 shows that flavone **D8** (quercetin) (Figure 2(D)) is a competitive inhibitor while the very similar **D10** (Figure 2(E)) is non-competitive. The predicted binding mode for flavones **D8** (quercetin) and **D10** brings the B-ring into the small reactive catalytic pocket formed by the Asp349/Glu276/Asp214 triad. The 3′-OH of flavone **D8** (quercetin) makes two hydrogen bonds with Glu276, while **D10**, which is slightly rotated, accepts one hydrogen bond from Asp214 and donates another to Asp349 (Figure 3(E,F)). The 4′-OH of flavone **D8** (quercetin) hydrogen bonds to Asp214 while 4′-OH from **D10** hydrogen bonds to Asp68. The crucial difference between **D8** (quercetin) and **D10** seems to be the C-ring substitution. The 3-OH group in flavone **D8** (quercetin) is able to hydrogen bond the conserved Asp349, while the hydrophobic 3-Cl-substitution in flavone **D10** cannot provide any specific interaction. The lack of this interaction makes **D10** to rotate and changes slightly the hydrogen bond pattern found for 3′,4′-(OH)_2_ substitution.

The extensive hydrogen bonding with the deep reactive pocket of flavone **D8** (quercetin) is absolutely consistent with the competitive inhibition pattern seen in Figure 2(D). **D10**, in contrast, loses the hydrogen bonds with one of the Asp349/Glu276/Asp214 triad members (namely with Asp214), weakening the interaction with the deep reactive pocket, also very well in line with the non-competitive pattern shown in Figure 2(E). Therefore, extensive hydrogen bond with the Glu/Asp triad seems to be relevant to hold the inhibitors bound to the deep reactive pocket.

#### Flavanone E3 (taxifolin)

Flavanone **E3** (taxifolin) contains 3′,4′-(OH)_2_ substitution in the B-ring, which were demonstrated to be important to drive the B-ring into the Glu276/Asp214/Asp349 triad pocket^32^. However, the lack of C2 = C3 double bound makes the B-ring to move out of the plane of the A- and C-rings, destroying the overall planarity of the compounds, which has a major impact in the binding properties. The predicted binding pose of **E3** (taxifolin) does not have the B-ring inserted in the small active site pocket. Instead, the entire flavanone moves to a larger, less deep pocket (Figure 3(G)). This result is consistent with the non-competitive mode of inhibition determined through the Lineweaver–Burk double reciprocal plots of Figure 2(F). The B-ring binds a hydrophobic pocket formed by Phe157, Leu176, Phe177, Thr215, Leu218, Ala278 and Phe300, and with 3′-OH hydrogen bonding to Glu276. The 5-OH of A-ring hydrogen bonds to the backbone of Asp349 and 7-OH of the same ring hydrogen bonds to the Phe157 backbone.

## Discussion

In this work, we explored a panel of flavonoids, covering a solid set of different structures, tested in the same model, under the same experimental conditions, which enabled us to draw relevant conclusions about the relationship structure *versus* activity.

Many of the studied flavonoids offer a promising alternative for the management of PPHG, presenting an IC_50_ ≤ 200 μM, much lower than the one found for **acarbose** (607 ± 56 μM), the positive control.

We started our study by testing a flavone without –OH groups (**A1**), which was not able to inhibit α-glucosidase. We also tested flavonoids **A2**, **A3** and **A4**, and all of them showed low or no inhibitory activity. From A group, the most active flavonoid was **A5**, which has a 3-OH in the C-ring and presented an IC_50_ of 54 ± 3 µM. The replacement of the –OH groups of the catechol in B-ring by –OMe groups (**A6**) decreased the inhibitory activity of the flavonoid.

The importance of the –OH groups position was also highlighted when A5, with OH groups at 3-position of C-ring and at 3′- and 4′- positions of B-ring, was tested, because the obtained IC_50_ was lower than the one obtained for A5, with –OH groups at 5 position of A-ring and at 3′- and 4′- positions of B-ring.

We followed our rationale of increasing the complexity of the structure, by the addition of a catechol group in the A-ring. The most effective compound was **C7**, studied here for the first time, with an IC_50_ = 7.6 ± 0.4 µM. The presence of two catechol groups distributed in the A and B rings, together with a 3-OH group in the C-ring, seems to be the more favourable structure for the inhibition of the enzyme. In fact, and as it can be seen in Table 1, the other related compounds (**C1**–**C6**) that differ from **C7** by the lack of an –OH group in some positions, have considerable weaker activities. Gao et al.^27^ corroborate our results showing that **B1** and **C1** have no activity against α-glucosidase catalytic activity. In the present study, we observed that the addition of a catechol group to the B-ring in both compounds increased their activity, the –OH group in 5-position of the A-ring (**B4**) being better than in 7-position (**C6**) (IC_50_ = 66 ± 7 µM and IC_50_= 95 ± 7 µM, respectively). The obtained results also have shown that, as mentioned by Gao and co-workers^27^, baicalein has a favourable structure for the intended effect, as observed in the present study for the same flavonoid [**D3** (baicalein)], which presents an IC_50_ of 44 ± 3 μM. Nevertheless, and corroborating our results, the activity increased substantially with the hydroxylation at the B-ring. In addition, comparing the results obtained for **C4** and **C5** it is possible to infer that the presence of the –OH group at the 3′-position (**C4**) is more relevant than at the 4′-position (**C5**) of the B-ring, as **C4** is more active than **C5**. In turn, with the basic structure studied by Gao et al.^54^ (5,6,7-trihydroxyflavone), only the 4′-hydroxylation of the B-ring enhanced the inhibitory activity of the 5,6,7-trihydroxyflavone. We can also conclude from the obtained results that the addition of hydrophobic and bulky substituents in the flavonoid structure decreases its activity, as it was possible to see in flavonoids that possess –OMe and/or –OBn groups (**C8**–**C13**).

We also tested another group of flavonoids, based on chrysin (**D1**) structure, in the D group. Once again, our results are in agreement with Gao et al.^54^, since chrysin had no inhibitory effect against α-glucosidase activity. Hydroxylation at 4′-position of the B-ring of chrysin (**D1**), giving apigenin (**D5**), increased the activity reaching an IC_50_ = 82 ± 6 µM. With this basic structure, the presence of an –OH group at 3′- (**D4**) or 4′- (**D5**) positions of the B-ring seems to be indifferent for the inhibitory activity.

It has been postulated that –OH groups in the B-ring are favourable for the inhibitory activity of the compounds^16^ ^,^ ^31^ ^,^ ^32^. Zeng et al.^24^ compared the activity of morin and myricetin and concluded that myricetin, that has three –OH groups at the 3′-, 4′- and 5′-positions of the B-ring, was more potent than morin that has only two –OH groups at 2′- and 4′-positions. Comparing **D6** (kaempferol) and **D8** (quercetin) or **D5** (apigenin) and **D7** (luteolin), it is possible to conclude that the presence of the catechol group in the B-ring increased considerably the activity of the flavonoids. Additionally, our results indicate that **D8** (quercetin) is a stronger inhibitor when compared with **D9** (morin). These findings clearly demonstrate that the *ortho* –OH groups might be responsible for the higher inhibitory effect, which seems to promote the interaction between α-glucosidase and flavonoids, while the *meta*-position of –OH groups decreases the electron cloud density of band I resulting in a lower inhibitory activity^24^ ^,^ ^32^. Comparing the activity of **D8** (quercetin) (IC_50_ = 15 ± 3 μM) and **D7** (luteolin) (IC_50_ = 46 ± 6 μM), we can conclude that the presence of a 3-OH group in the C-ring increases the ability to inhibit α-glucosidase. This result is corroborated by Nicolle et al.^55^ and Xu^32^ who suggested that the 3-OH in the C-ring may have the ability to make flavonoids bind into the binding pocket of yeast α-glucosidase properly to maintain a high inhibitory activity.

It is currently accepted that modification of any of the –OH groups in the A-ring diminishes the α-glucosidase inhibitory effects of flavonoids^27^ ^,^ ^56–58^. From the D group analysis, it becomes clear that **D3** (baicalein), which has –OH groups at 5-, 6- and 7-positions of A-ring, is more effective than **D2** (galangin) and **D1** (chrysin), with only –OH groups at 5- and 7-positions. These results are in agreement with Gao et al.^27^, that studied parent compounds of baicalein and reported that removal of any –OH group from the 5-, 6- and 7-positions led to a dramatic loss of the inhibitory potency. Moreover, here we concluded that the 5-hydroxylation at the A-ring is important for the intended effect, since the IC_50_ of **D6** (kaempferol) is almost three times lower than the IC_50_ of **C5**. Wang et al.^56^ results validate this finding, as compounds that bear one acetyl group at the 5-position of the A-ring of chrysin almost induce the disappearance of the flavonoids’ inhibitory activity. It is known that hydrogen bonding is a crucial factor for the interactions between the enzyme and its substrates and for the conformation and orientation of the inhibitors in the active site^8^. Kumar et al.^57^ showed that a 5-OH group of the A-ring acts as a hydrogen bond donor and consequently enhances the inhibitory activity of the flavonoid.

Our results illustrate that the presence of a 3-OH group in the C-ring is also relevant, evidenced by the lowest IC_50_ of **D5** (apigenin) and **D8** (quercetin) when compared with **D6** (kaempferol) and **D7** (luteolin), respectively. Accordingly, Silva et al.^25^, also compared the effect of luteolin and quercetin and suggested that 3-hydroxylation of the C-ring increases the inhibitory activity against α-glucosidase. Furthermore, Xu^32^ gathered information about the interaction of flavonoids with α-glucosidase, analysed with Autodock, and reported that the 3′,4′-(OH)_2_ groups of the B-ring together with a 3-OH group in the C-ring are crucial for the inhibitory effect of flavonoids.

In the panel of flavonoids in this work, we tested a series of chlorinated flavonoids (**D10**–**D14**) for the first time. It has been postulated that under diabetic conditions, the inflammatory process is exacerbated and consequently there is an excessive production of reactive species, namely, HOCl^59^. It is currently known the reactivity of flavonoids with HOCl in order to form stable mono and dichlorinated products^36^. Our group has recently shown that chlorinated flavonoids were more efficient than their parent compounds in modulate human neutrophils’ activities, foreseeing a potential anti-inflammatory activity^36^. These results raised our attention to these promising compounds, exploring here for the first time, their inhibitory effect against α-glucosidase. The chlorinated flavonoid **D10** was one of the most active compounds tested in this work, presenting an IC_50_ similar to the one obtained for **D8** (quercetin). Moreover, comparing compounds **D10** and **D8** (quercetin) it is possible to conclude that the substitution of a 3-OH group or a 3-Cl atom at the C-ring is almost indifferent for the inhibitory effect. In contrast, the results obtained with **D18** (rutin), reveal that the addition of a larger group, in this case rutinose, at the 3-position of the C-ring, weakens the activity of the flavonoid^16^.

Following the order of potency of the chlorinated flavonoids, it is possible to see that the second most effective compound was flavonoid **D14**, with –Cl atoms at 6 and 8 positions of the A-ring. However, when we have only one –Cl atom at 6 (**D11**) or 8 (**D12**) position of the A-ring, the inhibition of the enzyme decreased considerably.

We also tested a group of methoxylated compounds (**D15**–**D17**), and, corroborating the results obtained in A and C groups, the inhibitory effect on α-glucosidase was reduced by the introduction of an –OMe group, independently of the ring. According to the literature, the contribution of –OMe groups to the inhibitory effect of flavonoids depends on the basic structure of the flavonoid and/or with the type of substrate used^60^. Azuma et al.^60^ indicated that the α-glucosidase inhibition was enhanced by the presence of 5-OMe groups at the A-ring, and at 3′-, 4′-positions of the B-ring, when compared with quercetin. In turn, Gao et al.^54^ reported that, in contrast to –OH, the –OMe group substitution on the B-ring of 5,6,7-trihydroxyflavone was unfavourable for the activity.

It is known that the C2 = C3 double bond of flavonoids is crucial for their anti-inflammatory activity^37^ ^,^ ^61^ ^,^ ^62^. Analysing E group and comparing the inhibitory effect with some flavonoids of D group, namely, **D5** (apigenin) with **E1** (naringenin), **D7** (luteolin) with **E2** (eriodictyol), **D8** (quercetin) with **E3** (taxifolin), it is clear that the lack of C2 = C3 double bond weakens the flavonoids activity. This finding is in agreement with other reports about this issue^31^ ^,^ ^57^. Xiao et al.^15^ reported that the C2 = C3 double bond increases the π-conjugation of the bond linking the B and C rings, which favours near-planarity of the two rings. It is known that the molecules with near-planar structure easily enter the hydrophobic pockets in enzymes and subsequently can increase their inhibitory effect.

Once defined the effectiveness of the tested flavonoids, it was our aim to evaluate the type of α-glucosidase inhibition of the most active flavonoids from each group (A–E): **A5**, **B3**, **C7**, **D8** (quercetin) and **E3** (taxifolin). We also analysed the chlorinated flavonoid **D10**, since it was one of the most effective compounds, presenting an IC_50_ value similar to the one found for **D8** (quercetin). Lineweaver–Burk double reciprocal plots were employed to investigate the inhibition kinetics of the tested compounds against α-glucosidase activity. As observed in Figure 2, flavonoids **A5** (Figure 2(A)) and **B3** (Figure 2(B)) presented a mixed inhibition type (*K*_m_ value increased and *V*_max_ value decreased), which means that binding of the substrate or the inhibitor affects their enzyme binding affinity. In the mixed type inhibition, the inhibitor did not bind to the enzyme in the active site and both inhibitor and substrate can simultaneously be attached to the enzyme. However, the binding affinity for the substrate is decreased when the inhibitor is present. Concerning flavonoid **C7**, Figure 2(C) cannot clearly differentiate between a mixed and a competitive inhibition type. However, the interception of lines is close to the *y* axis. Moreover, the result obtained by docking calculations for flavone **C7** suggested that the binding pose prediction was directed to the active site. Considering the *in vitro* and docking studies, the results indicate that **C7** presents a competitive inhibition type. Flavonoid **D8** (quercetin) (Figure 2(D)) and **acarbose** (Figure 2(G)) are also competitive inhibitors, since the *K*_m_ value increased and the *V*_max_ value remained constant. As shown in Figure 2, flavonoids **D10** (Figure 2(E)) and **E3** (taxifolin) (Figure 2(F)) are non-competitive inhibitors (*K*_m_ value remained constant and *V*_max_ value decreased), which means that they can bind with equal affinity to both the free enzyme and the enzyme–substrate complex. In this inhibitory mechanism, the inhibitor binds to the enzyme at a site that is distinct from the substrate binding site (active site of the enzyme). A non-competitive inhibitor binds to an enzyme whether the substrate is at low or high concentrations^63–67^. To the best of our knowledge, this is the first report stating the type of inhibition for the mentioned flavonoids, except for **D8** (quercetin), which was already reported as a mixed type inhibitor^16^ ^,^ ^31^ ^,^ ^32^.

In what concerns **acarbose**, we verified a competitive inhibition type, as observed by the studies of Kim et al.^68^ and Lopez et al.^69^. Interestingly, Son et al.^70^ referred that this compound has a mixed type of inhibition.

The *K*_i_ values can also be a useful tool to compare the activity of the compounds, being reflective of the binding affinity of the inhibitor for the enzyme. The tested flavonoids presented the following order of Ki values: **C7** < **D8** (quercetin) < **A5 **< **D10**  < **B3** < **E3** (taxifolin). These results show that flavonoid **C7**, followed by **D8** (quercetin), exhibit the strongest affinity for the enzyme.

Flavones **A5**, **D8** (quercetin) and **C7** share very similar interactions with the active site and mimic interactions found between the first glucose of **acarbose** and the active site pocket. Moreover, the predicted mode of binding can rationalise the trends found across the series of studied flavonoids, namely the importance of 3-hydroxylation in the C-ring and the catechol group in the B-ring. The 3-OH substitution provides a hydrogen bond to Asp349, while the catechol in the B-ring hydrogen bonds Asp68, Asp214 and Glu276. Similarly, the inhibitor **acarbose** presents hydrogen bond interactions with the same active site amino acids.

## Conclusions

**Figure 4.**
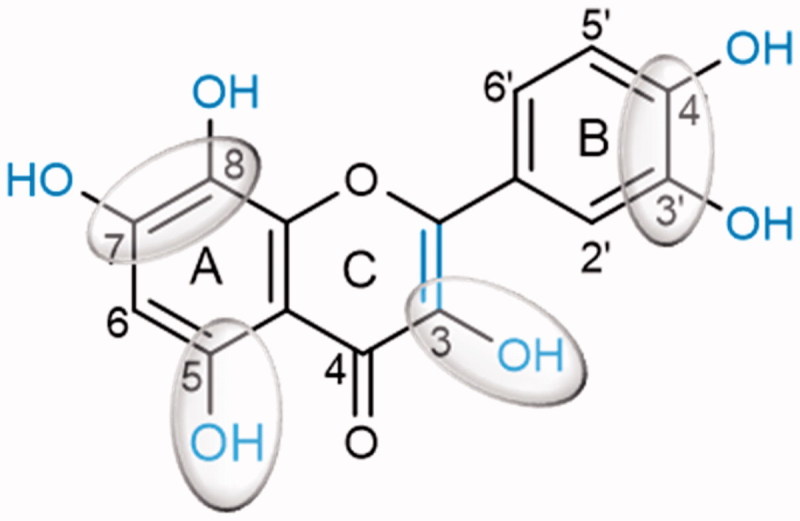
Potential flavonoids ‘substitution pattern that contributes to increase the α-glucosidase inhibition.

Our results have shown that adequately substituted flavonoids are effective α-glucosidase inhibitors. The flavonoid structure, the position and number of OH groups are determinant factors for the intended effect. The most active flavonoids were **C7** and **D8** (quercetin), which indicates that hydoxylations at 5- and 7- or 8-positions of the A-ring, at 3′ and 4′-positions of the B-ring and at 3-position of the C-ring, as well as the C2 = C3 double bond in the C-ring, are critical for the inhibitory activity of flavonoids (Figure 4). The results obtained in this study provide a series of potentially effective flavonoids that can be used as alternatives to the commonly administrated α-glucosidase inhibitors in the DM therapeutics.

## Supplementary Material

Supporting information
