# Supplementary material for: α-Glucosidase inhibition by flavonoids: an in vitro and in silico structure–activity relationship study
Source: J Enzyme Inhib Med Chem. 2017 Sep 21;32(1):1216–28. doi: 10.1080/14756366.2017.1368503 (PMC6009965; doi:10.1080/14756366.2017.1368503)
Supplement: Supporting information [file IENZ_A_1368503_SM8287.pdf]

Supporting Information

Model #01	File	Built with	Oligo-State	Ligands	GMQE	QMEAN
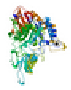	PDB	ProMod3 Version 1.0.2.	MONOMER	1 x CA: CALCIUM ION;	0.92	-0.20

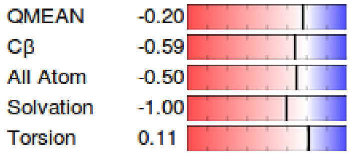

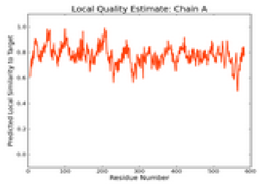

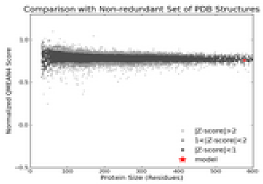

Template	Seq Identity	Oligo-state	Found by	Method	Resolution	Seq Similarity	Range	Coverage	Description
3aj7.1.A	72.51	monomer	BLAST	X-ray	1.30Å	0.54	6 - 582	1.00	Oligo-1,6-glucosidase

Ligand	Added to Model	Description
CA	✓	CALCIUM ION

Figure 1 –Summary of the homology modeling details for the template structure (3AJ7) and resulting *Saccharomyces cerevisiae* α-glucosidase homology model.

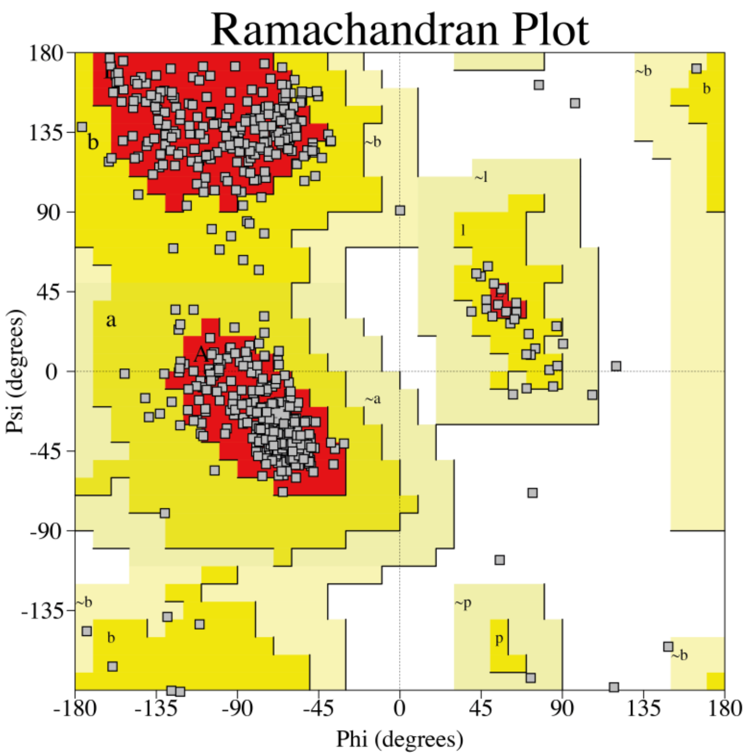

Figure 2-SI – Ramachandran plot analysis of *Saccharomyces cerevisiae* α-glucosidase homology model.

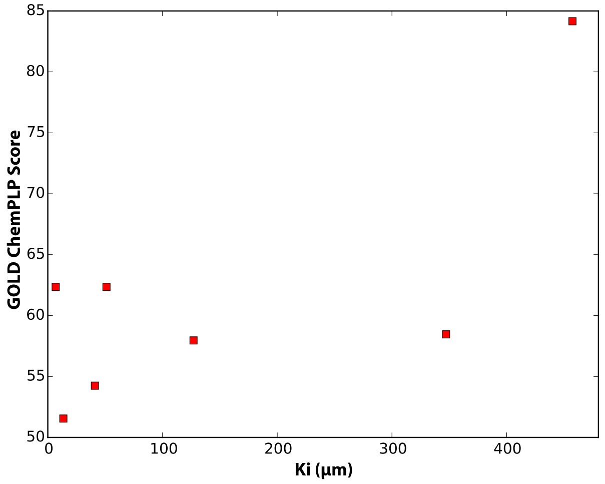

Figure 3-SI – Correlation between experimental Ki values and GOLD ChemPLP dimensionless score for A5, B3, C7, D8 (quercetin), D10, E3 (taxifolin) and acarbose.

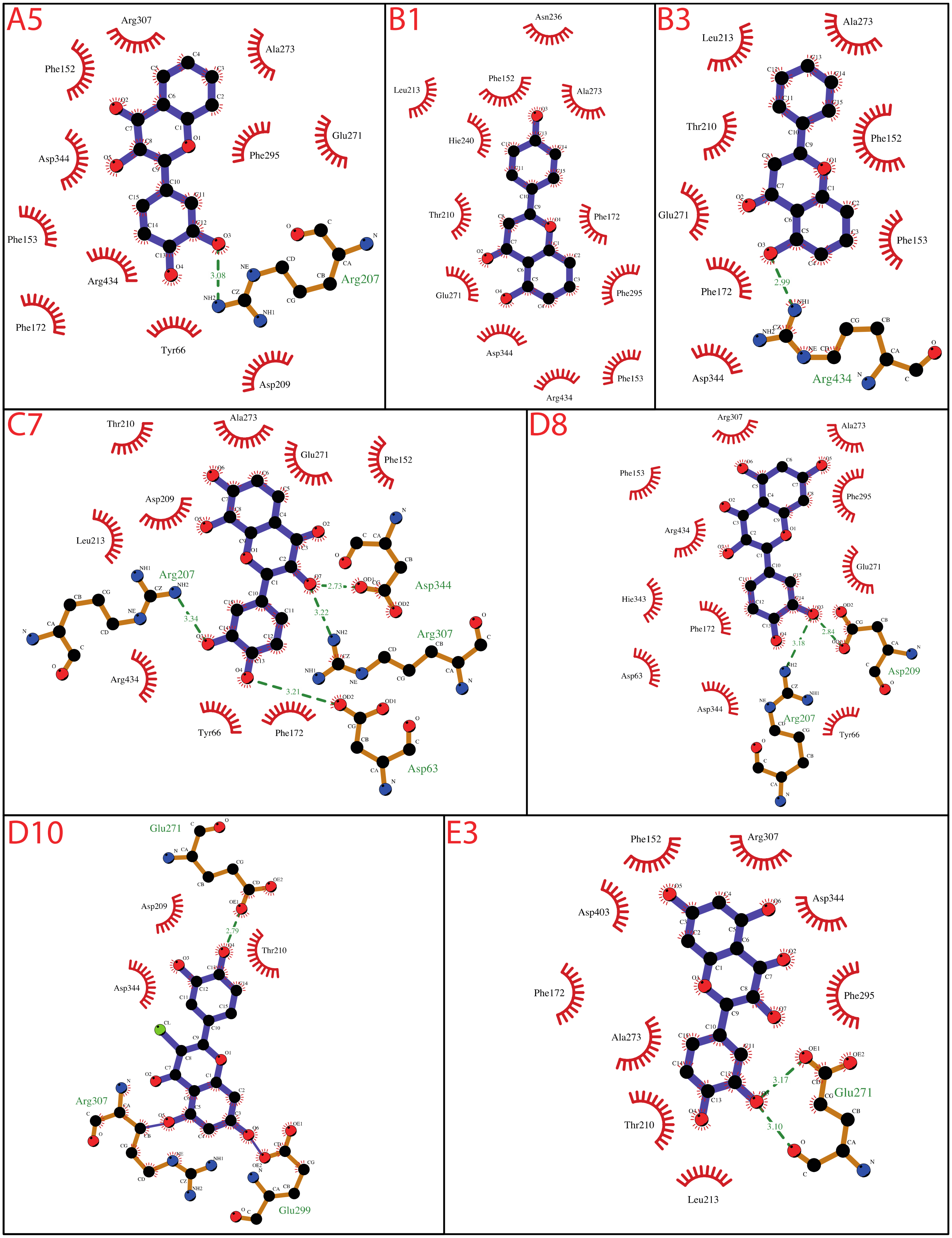

Figure 4-SI – Ligplot diagrams of the interaction between flavones A5, B1, B3, C7, D8 (quercetin), D10 and E3 (taxifolin) with *Saccharomyces cerevisiae* α-glucosidase homology model.

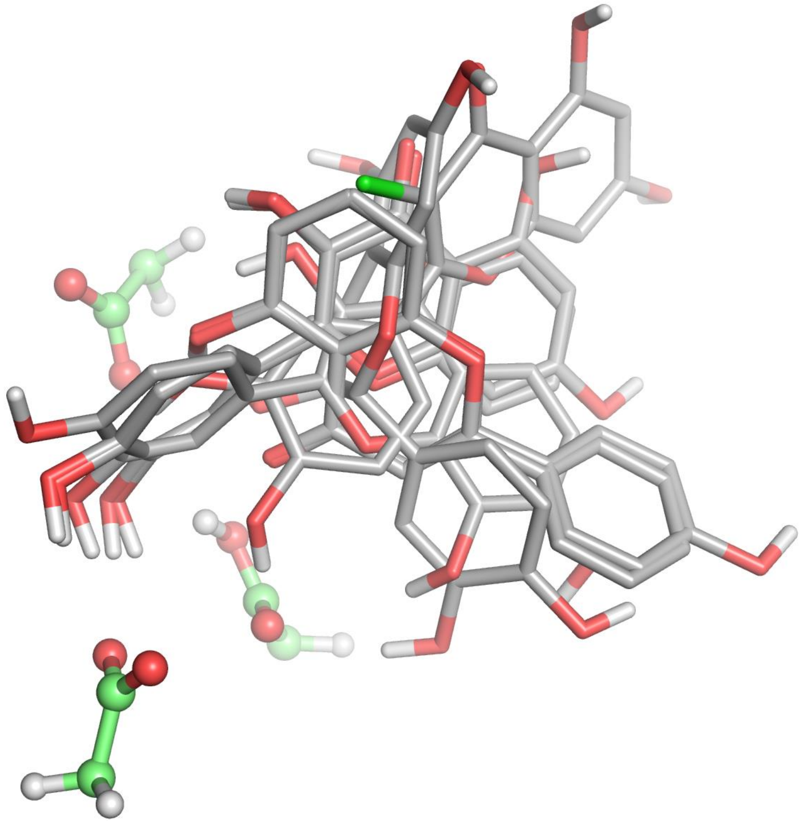

Figure 5-SI – Superimposition of the best docking solutions for compounds A5, B1, B3, C7, D8 (quercetin), D10 and E3 (taxifolin).

Protocol details for the system preparation, energy minimization and MD simulation of the generated homology model:

The system was solvated with TIP3P water molecules, in a rectangular box whose faces were at least 12 Å away from the protein. Non-bonded Coulomb interactions were treated with the particle Mesh-Ewald method and a cutoff of 10 Å was used for explicit short-range electrostatic and Lennard-Jones interactions. All bonds involving hydrogen atoms were constrained with SHAKE algorithm and an integration step of 2 fs was employed.

The geometry optimization was done in four steps: first, only the water molecules were minimized; afterwards, the hydrogen atoms were minimized; finally, in the third step, all but the backbone was fully minimized. MD simulations started by heating the systems for 50 ps, from 0 K to 310 K, in the NVT ensemble, followed by another 50 ps of NVT MD at 310 K. Subsequently we ran 2.5 ns in the NPT ensemble with the protein backbone still held fixed. Temperature and pressure were maintained at 310 K and 1 bar with the Langevin thermostat and the Berendsen barostat.

AutoDock Vina docking details:

Version: AutoDock Vina 1.1.2

center_x = 70.37, center_y = 42.15, center_z= 71.2, size_x = 15, size_y = 19.9, size_z= 17.3, num_modes = 9, energy_range = 3, exhaustiveness = 8
